# Biology and clinical relevance of follicular cytotoxic T cells

**DOI:** 10.3389/fimmu.2022.1036616

**Published:** 2022-12-14

**Authors:** Yuqi Lv, Laure Ricard, Béatrice Gaugler, He Huang, Yishan Ye

**Affiliations:** ^1^ Bone Marrow Transplantation Center, The First Affiliated Hospital of Zhejiang University School of Medicine, Hangzhou, Zhejiang, China; ^2^ Liangzhu Laboratory of Zhejiang University Medical Center, Hangzhou, Zhejiang, China; ^3^ Institute of Hematology, Zhejiang University, Hangzhou, Zhejiang, China; ^4^ Zhejiang Province Stem Cell and Cellular Immunotherapy Engineering Laboratory, Hangzhou, Zhejiang, China; ^5^ Sorbonne Université, INSERM, Centre de Recherche Saint-Antoine (CRSA), Paris, France; ^6^ AP-HP, Hôpital Saint-Antoine, Service d’Hématologie Clinique et Thérapie Cellulaire, Sorbonne Université, Paris, France

**Keywords:** follicular cytotoxic T cell, phenotype, cellular crosstalk, disease relevance, transcription factor

## Abstract

Follicular cytotoxic T (Tfc) cells are a newly identified subset of CD8^+^ T cells enriched in B cell follicles and their surroundings, which integrate multiple functions such as killing, memory, supporting and regulation. Tfc cells share similarities with follicular helper T (Tfh) cells, conventional cytotoxic CD8^+^ T (Tc cells)cells and follicular regulatory T (Tfr) cells, while they express distinct transcription factors, phenotype, and perform different functions. With the participation of cytokines and cell-cell interactions, Tfc cells modulate Tfh cells and B cells and play an essential role in regulating the humoral immunity. Furthermore, Tfc cells have been found to change in their frequencies and functions during the occurrence and progression of chronic infections, immune-mediated diseases and cancers. Strategies targeting Tfc cells are under investigations, bringing novel insights into control of these diseases. We summarize the characteristics of Tfc cells, and introduce the roles and potential targeting modalities of Tfc cells in different diseases.

## 1 Overview of Tfc cells

Follicular cytotoxic T (Tfc) cells are a CD8^+^ T cell subset initially discovered by Quigley et al. (1) in 2007. Tfc cells were found primarily inside and around the B cell follicles (2–4), while a small subset of Tfc cells localized in peripheral blood (1, 2). In addition, they were found in a variety of species, including mice, rhesus monkeys, and humans (5–7). Tfc cells are typically identified as CXCR5^+^ Tcf1^+^ Tim3^-^ CD8^+^ T cells, and their development is regulated by core transcription factors Tcf1, Bcl6, Blimp-1, E2a and Runx3. Meanwhile, Tfc cell differentiation is positively regulated by cytokines IL-21, IL-6, IL-23 and TGF-β. Since they maintain stemness and undergo a follicular development pathway, they share similarities with follicular helper T (Tfh) cells, follicular regulatory T (Tfr) cells, and newly identified “stem-like” CD8^+^ T cells. Furthermore, Tfc cells secrete cytokines IL-2, IL-4, IL-21, IFN-γ, TNF-α, granzyme B (Gzmb) and perforin under different conditions, and perform multiple functions such as killing, supporting and regulation. Participation of Tfc cells in chronic infections, immune-mediated diseases and tumors have been revealed. Changes in the frequency, phenotype and functions of Tfc cells affect the local immune homeostasis, which may mediate the pathophysiology and affect the severity of these diseases. In recent decade, great progress has been made in our understandings of Tfc cells. Deeper understandings on Tfc cell biology and their roles in diseases have shed light on potential therapeutic modalities targeting Tfc cells.

## 2 The characteristic surface markers of Tfc cells

**Figure 1:**
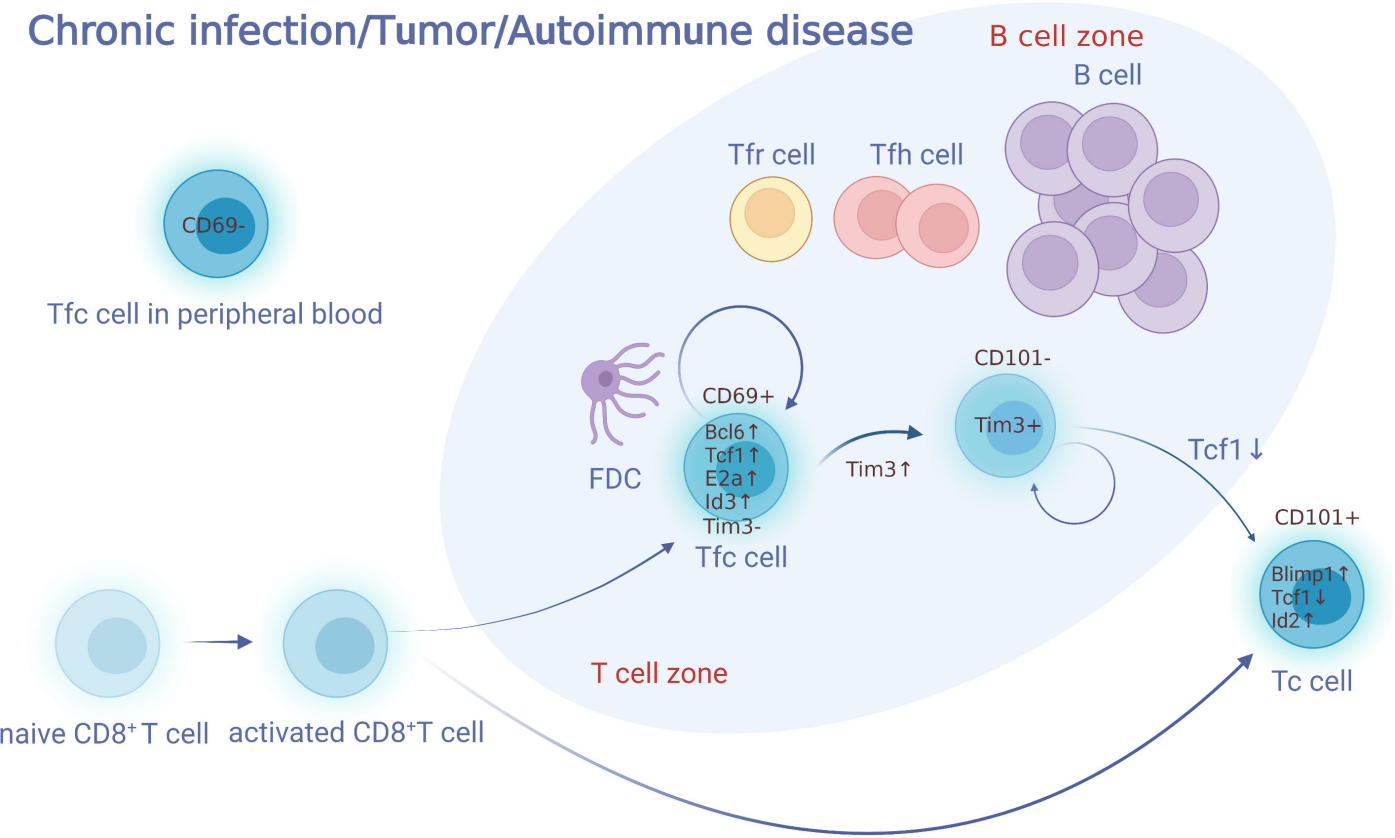
Development of Tfc cells. Naïve CD8^+^ T cells are activated and differentiated into activated CD8^+^T cells. A part of activated CD8^+^ T cells express CXCR5 and migrate to B cell follicles and their surroundings along the gradient of CXCL13 concentration. These cell subsets, defined as Tfc cells, perform various biological functions such as killing, memory, supporting, and regulation. Tfc cells do not express Tim3, and have the ability to self-renew. Tfc cells upregulate Tim3 and differentiate into two groups of Tim3^+^ CD8^+^ T cells (CD101^−^Tim3^+^ CD8^+^T and CD101^+^Tim3^+^CD8^+^T). CD101^−^Tim3^+^CD8^+^ T cells is a transitory cell population, which preserves proliferative and effector activity before transform into effector CD101^+^Tim3^+^CD8^+^ T cells irreversibly. Furthermore, Tfc cells present in both of the lymphoid organs and the peripheral blood, with those in the lymphoid organ expressing CD69 while those in the peripheral do not express. FDC, follicular dendritic cell; Tfh, follicular helper; Tc, cytotoxic T; Tfc, follicular cytotoxic T.

Tfc cells derive from CD8^+^ T cells which migrate towards GCs, and express signature markers of both CD8^+^ cytotoxic T cells and follicular T cells. CXCR5^+^ Tcf1^+^ Tim3^-^ CD8^+^ are classical surface markers to identify Tfc cells (8). Stromal cells and follicular dendritic cells (FDCs) secrete large amount of CXCL13, and construct an environment with both soluble and immobilized CXCL13 gradients (9, 10). CXCR5 is the corresponding receptor of CXCL13, which induces T cells to migrate toward B-cell follicles. Naïve CD8^+^ T cells are activated and differentiated into activated CD8^+^ T cells. A part of them express CXCR5 and migrate to B cell follicles and their surroundings along the gradient of CXCL13 concentration (**Figure 1**). In contrast to CXCR5, CCR7 is an important factor facilitating the migration of Tfc cells towards the T cell zone in response to CCL21, which is highly expressed in the T cell area. Se Jin Im et al. (5) observed that CXCR5^+^ CD8^+^ Tfc cells with high level of CCR7 mRNA expression resided in the T cell zone of mice spleen. Down-regulation of CCR7 led to the migration of Tfc cells from the T cell zone or T-B borders to the B cell zone (7). In addition, CXCR3, CD62L and CD69 are related to Tfc cell chemotaxis toward lymphoid tissue. CXCR3 is found on activated T cells and assists in their recruitment (11, 12). CXCR3 expression on Tfc cells is higher than that on naïve CD8^+^ T cells and Tc cells, which may facilitates their migration from peripheral blood toward infected B lymphocyte follicles (4, 8). Tfc cells express higher level of Sell and its encoded protein CD62L than Tc cells, indicating a memory phenotype and mediating lymphocyte adhesion and lymph node (LN)-homing of Tfc cells (4, 13). CD69 has been linked to the rapid activation of T cells during acute inflammation, and it has been shown to interfere with the function of S1P receptors, limiting S1P-mediated egress of immune cells from lymphoid organs into lymphatic vessels (8). Therefore, CD69^+^ Tfc cells reside in the lymphoid organs quiescently, whereas CD69^-^ Tfc cells remain in the peripheral blood.

45RO is highly expressed on Tfc cells, indicating that they may be derived from naive CD8^+^ T cells after contact with antigens (1). CD27, CD28 and CD69 are specific biomarkers on the early stage of T cell differentiation, which are also highly expressed on Tfc cells, suggesting Tfc cells as an early effector memory T cell subset. After antigen stimulation, Tfc cells elevate the expression of CD40L and ICOSL, which activates GC B cells *via* the CD40L/CD40 and ICOSL/ICOS, respectively (14).

Compared with Tc cells, Tfc cells express lower level of inhibitory molecules such as PD-1, GITR, CD244 and CD160 (3–6, 15–19). Tfc cells do not express Tim3, and have the ability to self-renew. Tfc cells upregulate Tim3 and differentiate into two groups of Tim3^+^ CD8^+^ T cells (CD101^−^ Tim3^+^ CD8^+^ T and CD101^+^ Tim3^+^ CD8^+^ T). In addition, CD8^+^ T cells up-regulate the PD-1 expression and develop status of cell exhaustion under the long and high-load antigen exposure, so the lower expression of PD-1 on Tfc cells might indicate a lower degree of cell exhaustion (3–5, 20). Notably, the expression level of PD-1 on Tfc cells varies among diseases and different levels of antigen stimulation. Compared with healthy individuals or NHL patients, PD-1 expression on Tfc cells is lower among CLL patients (5). Moreover, PD-1 expression on Tfc cells infiltrated in the tumor samples of follicular lymphoma patients is higher than that expressed on Tfc cells sorted from tonsil samples of healthy individual (15). Interestingly, upon specific condition such as allograft transplant, a subset of PD-1 negative Tfc cells have been observed to inhibit allo-antibody secretion from alloreactive B cells (21). Finally, given that PD-1 controls the localization of Tfh cells in both co-stimulus-independent and co-stimulus-dependent manners, it is also speculated that PD-1 may be also related to the localization of Tfc cells (15, 22–24).

## 3 Transcription factors involved in Tfc cells differentiation

**Figure 2:**
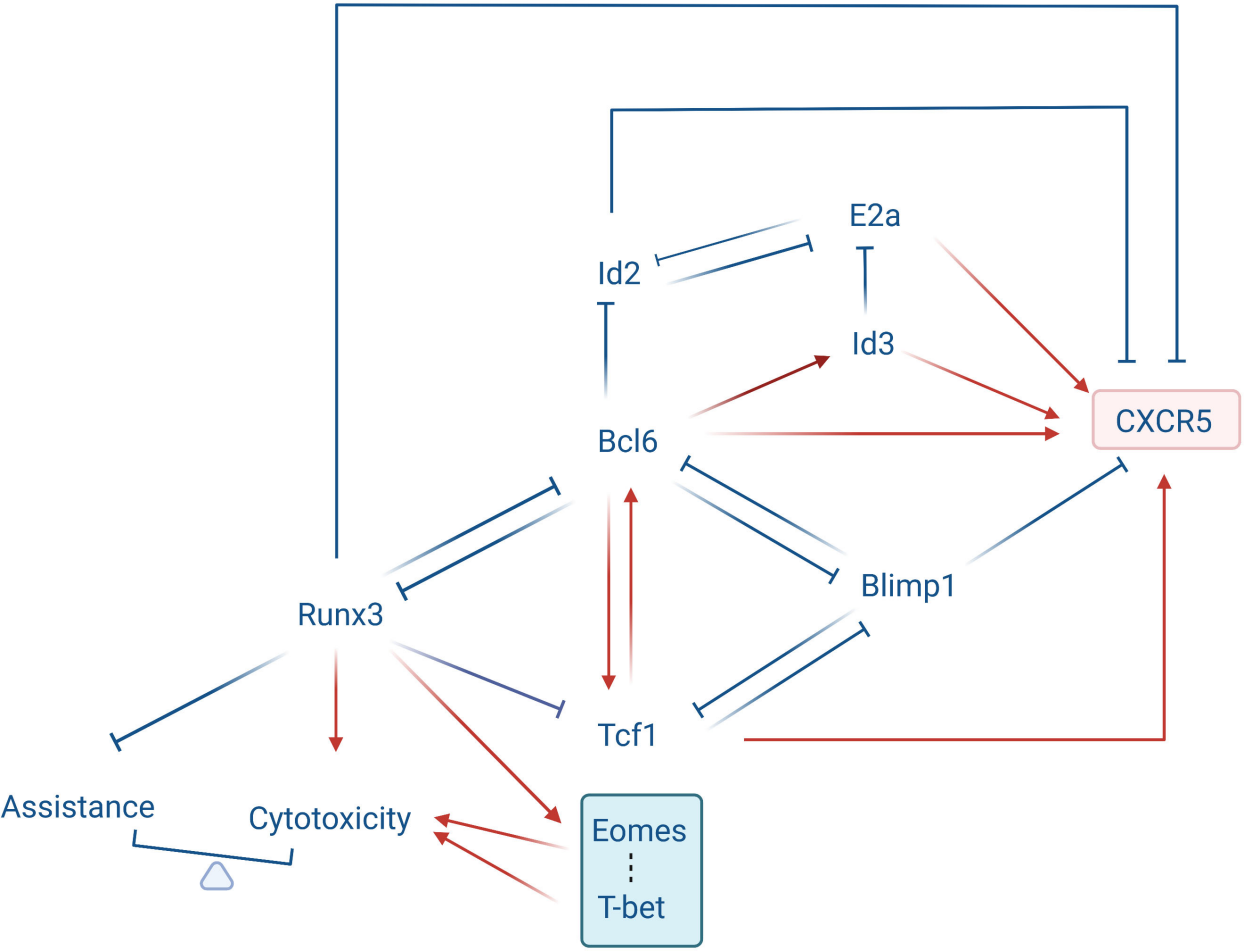
Transcriptional loop governing Tfc cell development. Bcl6, Tcf1, Eomes, and the E2a inhibitors Id2 and Id3 form a transcriptional loop that determines Tfc cell development. Tcf1 is highly expressed in Tfc cells and acts as an upstream regulator of the Bcl6-Blimp1 crosstalk, promoting Bcl6 expression while suppressing Blimp1 expression. Tcf1, Bcl6 and Blimp1 are mutually antagonistic or promoting. Bcl6 is highly expressed in Tfc cells and promotes CXCR5 protein expression while decreasing Runx3 and Id2 expression. Blimp1 is downregulated in Tfc cells, and it suppresses Cxcr5 gene transcription by binding to its 5’ upstream and intronic regions. E2a activation enhances Cxcr5 transcription and protein production through binding to its intron region. Id2, which antagonizes E2a transcriptional activity, suppresses Cxcr5 expression. Id3, however, a transcriptional antagonist of E2a, promotes the Cxcr5 expression. Runx3 is down-regulated in Tfc cells, it represses the Bcl6 expression and the Tfh program, and activates the cytotoxic program directly. In addition, Runx3 promotes the expression of T-box family transcription factors (T-bet and Eomes), which promotes the cytotoxicity of Tfc cells, while limiting the assistant capacity.

Bcl6, Tcf1, Eomes and E2a inhibitors Id2 and Id3 form a transcriptional control loop that together guide the development of Tfc cells (**Figure 2**). The differentiation of Tfc cells can be promoted by the TFs Bcl6, E2a, and Tcf1, and inhibited by Blimp1 and Id2 (4, 15). Bcl6 and Blimp1 are antagonistic TFs. Compared with CXCR5^-^ non-Tfc cells, Tfc cells express higher levels of Bcl6, while Blimp1 expression is significantly reduced. Cluster analysis has shown that a large number of differentials expressed genes which drive Tfc cell differentiation are Bcl6-bounded. The proportion of CXCR5^+^ CD8^+^ T cells among CD8^+^ T cells in Bcl6 overexpressed mice infected with LCMV increased significantly from 20% to 60%. After knocking down the *Bcl6*, CD8^+^ T cells failed to differentiate into Tfc cells *in vivo* on the 8^th^ day after infection with LCMV. In the transcriptional regulation aspects, up-regulation of Bcl6 enhanced the expression of *Tcf7* (encoding Tcf1) and *Id3*, while inhibited the expressions of *Prdm1* (encoding Blimp1) and *Id2*. Meanwhile, over-expression of Bcl6 resulted in most phenotypic changes on Tfc cells, including up-regulation of CD127, CD62L and ICOS, and down-regulation of Tim-3 (4). In contrary to Bcl6, Blimp1 inhibits Tfc cells differentiation. It was observed that in Blimp1 deficient CD8^+^ T cells the characteristic TFs of Tfc cells were upregulated, and the CXCR5 expression was significantly increased (4, 25). Finally, up-regression of Blimp1 in activated CD8^+^ T cells using retroviral vectors containing Blimp1-binding motifs has shown that Blimp1 suppresses CXCR5 promoter activition (4).

Tcf1 is a key transcription factor regulating the Bcl6-Blimp1 axis during Tfc cell development. Tcf1 induced Bcl6 expression and repressed several pro-exhaustion factors including Blimp1, Tim3 and Cish, which could repress T cell exhaustion and maintain T cell stemness (26). During chronic infection, Virus-specific Tcf1^high^ CD8^+^ Tfc cells differentiate into a less exhausted Tcf1^high^ CD8^+^ T cell subset and a more exhausted Tcf1^low^ CD8^+^ Tc cell subset, respectively. Here Tfc cells serve as progenitor-like subsets supplementing Tcf1^low^ CD8^+^ Tc cells, which is critical for persistent antiviral CD8^+^ T cell responses in chronic infection (27).

Compared to CXCR5^-^ CD8^+^ T cells, Tfc cells express lower level of runt-related transcription factor 3 (Runx3) (6). Runx3 modulates a broad transcriptional network. Owing to the down-regulation of Runx3, the major function of CD8^+^ T cells changes from cytotoxicity to B cell assistance (28). It is revealed that Runx3 enhances the cytotoxic function of Tc cells through *Prf1* and *Gzmb* binding, and its knockout leads Tc cells to differentiate into Tfc-like cells, which migrate into the B follicles and assist antibody production (28–30). In Runx3-difficient Tc cells, characteristic genes associated with follicular T cell linage including *Bcl6, Tcf7, cxcr5, Icos, il6ra and il21* are up-regulated. Meanwhile, GSEA plot has also shown gene expression similarities between Runx3^-/-^ Tc cells and follicular T cells (28). Finally, during acute infection Runx3 promotes Tc cell clonal expansion, and prevents activation of the Tfh program in CD8^+^ T cells through Tcf1 repression.

During Tfc cell development, During Tfc cell development, Runx3 induces the expression of T-box family TFs (30). Tfc cells only express two T-box family TFs: T-bet and Eomes, which antagonize with each other during the linage differentiation of CD8^+^ T cells (31–33). T-bet regulates cytotoxicity of CD8^+^ T cells by increasing perforin and IFN-γ secretion and promoting IL-2 and PD-1 expression (34–36). It is observed that Tfc cells express lower level of T-bet and higher level of Eomes as compared to Tc cells, and this Eomes^hi^ T-bet^low^ feature confers Tfc cells an early memory phenotype with lower cytotoxicity than their CXCR5^-^ counterparts (15).

Id2 and E2a antagonize each other not only in the CXCR5 expression but also the development of Tfc cells. Id2 inhibits the Cxcr5 expression and the Tfc cells differentiation. He et al. observed that on the 21^st^ day after LCMV infection, the number and frequency of Tfc cells in Id2^-/-^ mice were higher than those in wildtype group, and the virus titer in Id2^-/-^ mice was significantly lower. In Tc cells, E2a promotes CXCR5 expression *via* binding with a conserved E2a-binding sequence in *Cxcr5* intron region. Over-expressing E2a in LCMV-specific P14 CD8^+^ T cells remarkably upregulated CXCR5 expression and the frequency of CXCR5^+^ cells in P14 CD8^+^ T cells, whereas co-overexpressing Id2 compromised such effect. In addition, E2a overexpression is related to increased CD107 expression and cytokine secretions, and decreased PD-1 expression on P14 CD8^+^ T cells. Importantly, during chronic viral infection, the Id2/E2a axis plays a key role in driving the differentiation from virus-specific CD8^+^ T cells to CXCR5^+^ and CXCR5^-^ subgroups, performing anti-viral effect both inside and outside the follicles. It was observed that Tfc cells appear in chronic infected organisms and supplement CXCR5^-^ CD8^+^ T cells outside the follicles. Abundance of Id2 in Tfc cells help them to transform into CXCR5^-^ CD8^+^ T cells, which secrete more IFN-γ and TNF- compared to Tc cells originally reside outside the GCs (3). By contrast, down-regulation of Id2 has been found to transfer virus-specific exhausted CD8^+^ T cells towards Tfc cells and migrates into follicles (28). Interestingly, another E2a antagonize TF Id3 is highly-expressed in Tfc cells, indicating a potential self-limiting regulation of CXCR5 expression in Tfc cells through the Id3-E2a crosstalk. When *Id3* expression is disrupted, the CXCR5 expression on LCMV-specific Tfc cells was significantly increased. Meanwhile, over-expression of Id3 prevents CD8^+^ T cell transformation towards Tfc cells due to down-regulation of several genes relevant to Tfc development (4).

## 4 Cytokines related to Tfc cell development and functions

### 4.1 IL-21 and IL-6

IL-21 receptor (IL-21R) is expressed on Tfc cells, while the effects of IL-21 on Tfc development and functions remain elusive. It is observed that during chronic inflammation, activated CD4^+^ Tfh-like cells enter tissues, produce IL-21 and activate CD8^+^ T cells through JAK/STAT protein phosphorylation (37). Afterwards, CD8^+^ T cells release IFN-γ and change their metabolic profile. Target genes of the IL-21/IL-21R signaling include *Batf、Bcl6、Eomes、Gzma、Gzmb、Il10、Maf、Prdm1* (38, 39). In addition, IL-21 promotes the transcription of Id2, which down-regulates CXCR5 expression on CD8^+^ T cells (40, 41). It is possible that Tfc cells are also affected in a similar way, while more experimental evidence should be provided.

Tfc cells secrete IL-21, which promotes B cell maturation, antibody secretion and class-switch recombination *in vitro* (42, 43). Circulating Tfc cells in chronic HBV infection express IL-10 and IL-21, which enhance Tfh cell function and induce B cell antibody production synergistically (42). In addition, IL-21 secreted by Tfc cells may promote the development of HL by stimulating the IL-21R on the surface of R-S cells (44). Deficiency in Runx3 or STAT5 elevates the IL-21 secretion from Tfc cells (28). Down-regulation of STAT5 impairs the Blimp1 expression, and increases the expressions of Bcl6, Batf and IL-21. In some certain conditions, Tfc cells do not secrete IL-21. For example, Tfc cells in LCMV infected mice do not express ICOSL and IL-21, while they perform more potent cytotoxicity (4).

The IL-6 signaling is essential for Tfc cell differentiation at the early stage, which promotes IL-21 secretion through Stat3 up-regulation. Yang et al. (43) have found that IL-6R is an important marker to identify Tfc cell subsets producing IL-21, and an IL-6-rich microenvironment is necessary for naïve CD8^+^ T cells to develop into Tfc cells highly secrete IL-21. Notably, the effect of IL-6 is timely-dependent, since late stimulation of activated CD8^+^ T cells with IL-6 can no longer induce their IL-21 production (43).

### 4.2 TGF-β

When PBMCs are cultured with anti-CD3/CD28 *in vitro* together with TGF-β, the expression of CXCR5 is up-regulated. When TGF-β is added to the CD8^+^ T_RM_ cell culture system, CXCR5 expression of CD8^+^ T_RM_ cells was higher than those stimulated by IL-12 and IL-23 (6). In addition, IL-23 and TGF-β induce a higher expression of CXCR5 in naïve CD8^+^ T cells (15, 45). TGF-β and IL-23 down-regulate the expression of Prdm1 and Id2 and increase the expressions of Bcl6 and Id3 by Tfc cells. It is also found that TGF-β activates E2a and promotes CD8^+^ T cells to go through the follicular differentiation pathway. In the meantime, TGF-β induces the Foxp3 expression of CD8^+^ T cells by promoting the E2a and Foxp3 promoter binding (46–48). Finally, non-canonical pathway of TGF-β and interactions with other signals may be also involved in Tfc cell follicular differentiation (45).

## 5 Comparison of Tfc cells and relevant T cells

There are similarities and disparities between Tfc cells and other cell types (summarized in **Table 1**). Firstly, for Tfc and Tfh cells, both of them undergo similar follicular development pathways regulated by core transcription factors Bcl6, Blimp1 and Tcf1. Meanwhile, their developments are regulated by cytokines IL-21 and IL-6, and their surface markers (CXCR5, ICOS, CD40L, etc.) are similar. However, some differences have made Tfc cells distinct from Tfh cells. Tfc and Tfh cells belong to CD8^+^ and CD4^+^ T cell lineages, respectively. Tfc cells may stay at an earlier stage of differentiation than Tfh cells. According to Yu Di et al. (8), the stage of differentiation of Tfc cells are between Tscm and Tcm cells, which may explain that stemness exist in Tfc cells but not in Tfh cells. Moreover, not like Tfh cells, Tfc cells are capable of self-renew, and have the ability to develop into cells with a killing function before entering an exhausted state. Tfc cells secrete cytokines IL-2, IL-4, IL-21, IFN-γ, TNF-α, Gzmb and perforin under different conditionsm, while Tfh cells secrete IL-6, IL-10, IL-21 to help them serve as B cell helpers. Tfc and Tfh cells can help B cells through CD40L/CD40, ICOSL/ICOS, TCR/MHC, as well as the cytokine IL-21 (2).

**Table 1:** Comparison among Tfc cell and other T cell subsets.

Cell subsets			Naïve CD8^+^ T	Stem-like CD 8^+^T	Tfc	Tc	Tfh	Treg	Tfr
**Lineage**		CD4	–	–	–	–	+	+	+
		CD8	+	+	+	+	–	–	–
**Surface marker**	**Migration**	CCR5			+	+	–		
		CCR7	+		+/-	–	–		–
		CXCR5	–	+	+	–	+	–	+
		CXCR3	+	+	+	+	+	+/-	
		CD62L	+	+	+/-	–	–	–	
	**Activation**	CD69	–	+/-	+/-	+	+	+	–
		CD27		+	+	+			
		CD45RA	+	–	–	–	–	–	–
		CD45RO	–	+	+	+	+	+	+
	**TNF superfamily**	CD40L			+	+			–
		FasL			+	+			
	**Co-stimulating molecules**	CTLA-4		–	+	+		+	+
		PD-1	–	+	+	+	+	+	+
		CD28	+	+	+	+			–
		ICOS	–	+	+	+	+	+	+
	**Others**	CD107	–		+	–			
		CD127	+		+	–	–		–
**Transcription regulator**		Bcl6	–	+	+	+	+	+	+
		Blimp1	–	+	+	+	+	+	+
		Tcf1	–	+	+	–	+		+
		Runx3			+		+		
		Tim3		–	–	+			
		T-bet	–	+	+	+	+	+	
		Eomes	–		+	–	+/-	+	
		Id2	–		+/-	+	–		+
		Id3	**+/-**		+		+		+
		E2a			+	+	+		+
		Stat3			+	**+/-**	+	+	+
		Maf			+		+	+	
		Batf			+		+	+	
		Foxp3	–	–	–	–	–	+	+
**Cytokines required for differentiation *in vitro***			–	–	IL-23, TGF-β, IL-6, IL-21	IL-2, IL-4, IL-6, IL-12, IL-21, TGF-β	IL-6, IL-12, TGF-β, IL-21, IL-23	IL-2, TGF-β	
**Cytokines secretion**			–	–	IL-21, IL-10, IFN-γ, perforin	IL-4, IL-5, IL-9, IL-10, IL-13, IL-17, IL-21, granzyme, perforin, TNF-α, TGF-β, IFN-γ, GM-CSF,	IL-4, IL-10, IL-17A, IL-17F, IL-21, CXCL13, IFN-γ, TNF-α	IL-9, IL-10, IL-35, TGF-β, CLL3, CLL4	IL-10, TGF-β, granzyme

+, positive; -, negative; +/-, either positive or negative, context-dependent.

Recent studies have described a specific subset of CD8^+^ T cells namely “stem-like” CD8^+^ T cells, which significantly overlap with Tfc cells in transcription factors, phenotypes, and functions (49–53). “Stem-like” CD8^+^ T cells express CXCR5 throughout their development but not when they develop into mature couterparts. Therefore, CXCR5 was the key surface marker for distinguishing this subset of “stem-like” CD8^+^ T. However, recent studies have identified Tcf1 as a more prominent marker than CXCR5 for Tfc cell identification. Tcf1-mediated Bcl6 induction and Blimp1 repression constitute crucial regulatory circuits in promoting “stem-like” CD8^+^ T cell fate, as well as regulated Cxcr5 expressions (4). Notably, “stem-like” CD8^+^ T cells and Tfc cells share many similarities, including the high expressions of Tcf1, CXCR5, ICOS, CD28, and weak expression of surface markers related to the cell depletion. In addition, both of them have the ability to self-renew and transform into more cytotoxic and exhausted T cells. However, Tfc cells not only exhibit “stem-like” features and serve as a CD8^+^ T storage pool, but they also eliminate Tfh cells and B cells, execute B cell helper functions, regulate B cells, and play a role in anti-tumor, antiviral, and autoimmune diseases. Collectively, Tfc cells seem to perform broader range of functions than “stem-like” CD8^+^ T cells, but it remains unclear whether “stem-like” CD8^+^ T cells represent a subset of Tfc cells, or “stem-like” CD8^+^ T cells are indeed Tfc cells with function partially revealed.

Tfc and Tfr cells are both important members of the humoral immunity. There are similarities and differences between Tfc and Tfr cells on transcription factors, surface markers, cytokines and functions. Firstly, Tfc cell development is dependent on Tcf1, Bcl6, Id2, and Runx3, and the expression of Blimp1 is down-regulated. However, Tfr cell development is dependent on Bcl6, Foxp3, and the expression of Blimp1 is up-regulated. Tfc cell differentiation is positively regulated by cytokines IL-21, IL-6, IL-23, and TGF-β, whereas Tfr cell differentiation is negatively regulated by cytokines IL-2, IL-6 and IL-21 (54–56). Secondly, even though Tfc and Tfr cells are CD8^+^ and CD4^+^ T cell lineages respectively, they both express CXCR5, Bcl6, Tcf1, PD1, and ICOS. However, Tfc cells expressed memory T cell markers CD28, CD27, and CD62L, while Tfr cells expressed Treg cell markers CTLA4, GITR, and Foxp3. Thirdly, different from Tfc cells, Tfr cells release IL-10, barely express IL-4 and IL-21 and cytokines related to cytotoxicity such as IFN-γ, TNF-α, Gzmb and perforin. Finally, both Tfc and Tfr cells play a vital role in regulating Tfh cells and GC-B cells, but the mechanisms are different. Tfc cells exert its regulatory role through not only Tfh and B cell elimination, but also cytokine secretion (described in the next chapter). Tfr cells produce IL-10, TGF-β to suppress Tfh and GC-B cells directly (15). Meanwhile, Tfr cells produce IL-1R2 and IL-1Rα to inhibit Tfh cell activation, and suppress the expression of B7-1 and B7-2 on GC-B cells *via* CTLA4, which finally down-regulate GC-B cell stimulation (57).

## 6 Crosstalk among Tfc, Tfh and B cells

**Figure 3:**
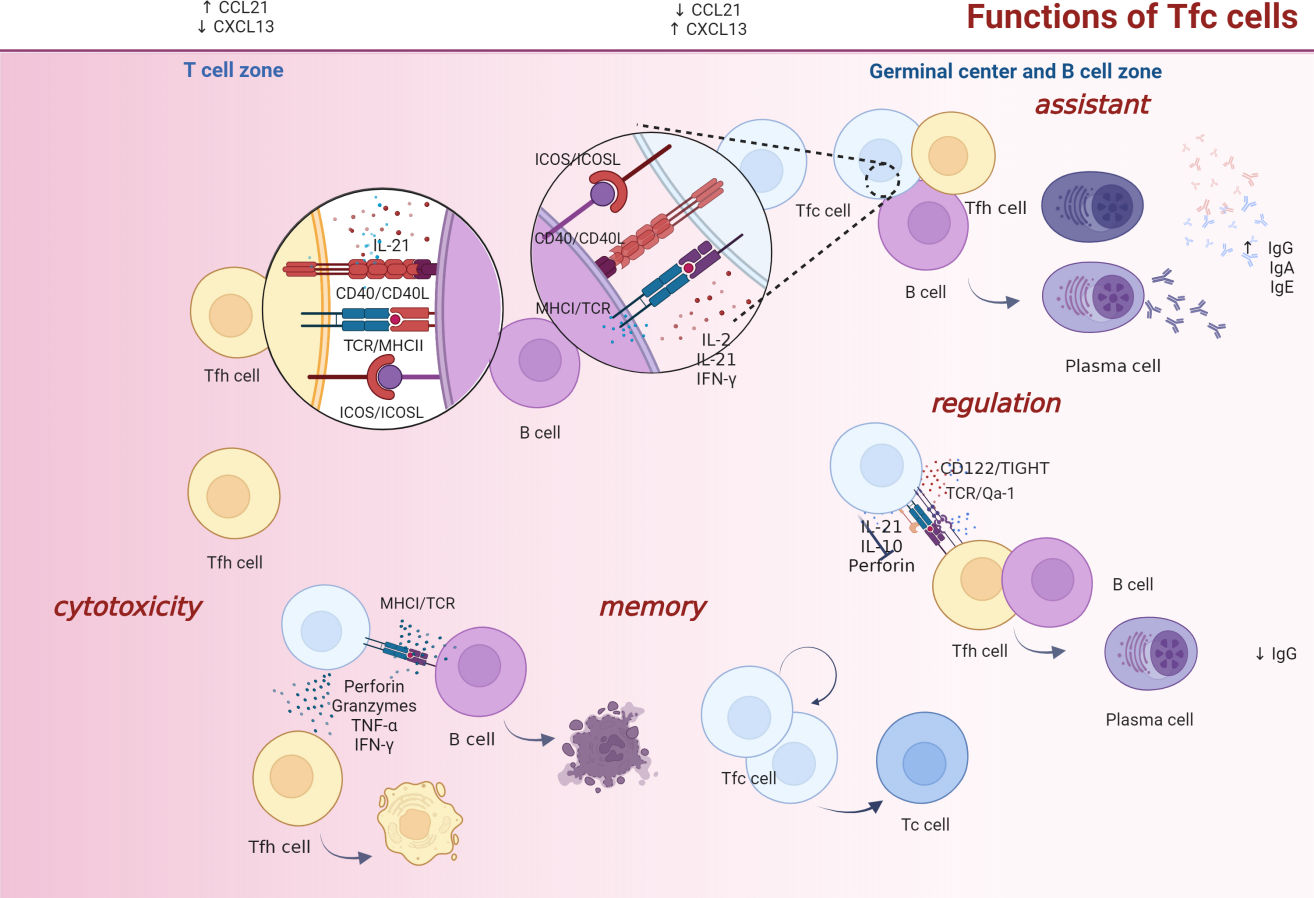
Tfc/Tfh/B cell interactions. Tfc cell functions are categorized as cytotoxic, memory, assistance, and regulatory. Firstly, Tfc cells perform their killing functions mainly through their cytokine secretion. They secrete IFN-γ, TNF-α, granzymes and perforin to eliminate infected Tfh and B cells. Secondly, under chronic infection settings, Tfc cells serve as a CD8+ T cell storage pool, which maintain stemness and differentiate into effector CD8+ T cells. Thirdly, Tfc cells help B cells produce antibodies and antibody isotype class switching *via* cytokines IL-2, IL-21, IFN-γ or with the help of CD40L/CD40, ICOSL/ICOS, and TCR/MHC I interactions. Fourthly, under a perforin-dependent and IL-10-independent condition, Tfc cells suppress Tfh cell helper function and antibody response through ICOSL/ICOS, CD122/TIGHT and TCR/Qa-1, and inhibit antibody responses to sustain self-maintenance and suppress immunity. Moreover, the IFN-γ, TNF-α, and granzymes A/B/K expression of Tfc cells increase, inhibit Tfh cell function in a cell-cell contact independent manner, and inhibit Tfh-dependent plasma blast cell differentiation indirectly. FDC, follicular dendritic cell; Tfh, follicular helper; Tc, cytotoxic T; Tfc, follicular cytotoxic T.

Functions of Tfc cells can be divided into four categories: cytotoxicity, memory, B cell antibody class-switch facilitation and B cell function enhancement (literatures summarized in **Table 2**). The common anatomical location provides a good communication space for Tfc, Tfh and B cells (crosstalk among these three types of T cell is illustrated in **Figure 3**). Cytotoxicity of Tfc cells manifests as removal of the infected/cancerous Tfh cells or B cells. Tfc cells secrete IFN-γ, TNF-α and granzymes to eliminate infected Tfh and B cells, and the number of Tfc cells was inversely correlated with viral load. In patients with chronic hepatitis B virus (HBV) infection and HBV-infected hepatocellular carcinoma, frequency of Tfc cells in the peripheral blood is significantly up-regulated, which is negatively correlated with frequency of Tfh cells (2). Besides, Tfc cells are less exhausted than CXCR5^−^ CD8^+^ T cells during chronic infection, and they serve as a CD8^+^ T storage pool and differentiate into terminally exhausted CD8^+^ T cells.

**Table 2:** Tfc cell functions and their settings.

Reference	Tfc definition	Function	WT/KO	Disease	Setting*in vitro*	Setting *in vivo*	Function
(58)	CD3^+^CXCR5^+^CD45RA^-^CD8^+^ T	Tfc cells secrete higher levels of IFN-g, IL-2, TNF and IL-10 than non-Tfc CD8^+^ T cells.	–	non-small cell lung cancer	Human tumor Infiltrating Tfc cells	–	cytotoxic
(59)	CCR7^lo^CXCR5^hi^CD27^hi/lo^CD45RO^hi^ CD8^+^ T	Tfc cells show good cytolytic potential characterized by high expression of granzyme B and perforin. Tfc cells with potent cytolytic activity are recruited to GCs during HIV infection and kill HIV infected cells.	-	HIV	Tfc cells from LN of HIV+ humans	-	cytotoxic
(60)	SIV-specific CD8^+^ T cell	SIV-specific CD8^+^ T cells restrict productive SIV infection to Tfh cells in elite controller monkeys.	–	SIV	–	SIV elite controllers/SIV typical progressors in rhesus monkey model	cytotoxic
(4)	CXCR5^+^CD200^+^ICOS^+^PD-1^+^Tcf1^+^Tim3^-^CD8^+^ T	Tfc cells control viral infection in Tfh cells; the frequency of LCMV-infected Tfh cells in mice that have received Cxcr5^–/–^ P14 cells is about twofold higher than that in mice receiving Cxcr5^+/+^ P14 cells. Tfc cells control viral infection in B cells; the frequency of MuHV-4-infected B cells is about 4.5-fold higher in mice that have received Tc cells than in mice that have received Tfc cells.	-	HIV, LCMV, murid herpesvirus 4	-	LCMV-infected mice; MuHV-4 infected mice	cytotoxic
(3)	CXCR5^+^CD44^hi^ICOSL^-^CD8^+^ T	Tfc cells are less exhausted than CXCR5^−^CD8^+^ T cells and control viral load during chronic infection. Upon stimulation with the indicated peptides, the IFN-γand TNF- αproductions from Tfc cells are higher than CXCR5^−^CD8^+^ T cells.	Cd4^Cre^ transgenic, μMT and C57BL/6J	Acute and chronic LCMV infection	–	CXCR5^+^CD44^hi^CD8^+^ T cells were adoptively transferred into Cl13-infected CD4^+^ T-cell-depleted recipients after LCMV infection.	cytotoxic
(61)	CCR7^lo^CD95^hi^CXCR5^hi^CXCR3^hi^CD8^+^T	Tfc cells have cytolytic potential and can be redirected to target and kill HIV-infected cells.	-	chronic SIV	Tfc cells from chronically SIV-infected rhesus macaques	chronically SIV-infected rhesus macaques	cytotoxic
(62)	CXCR5^+^PD-1^+^ICOS^+^CD40L^+^CD45RO^+^CD27^+^CCR7^-^CD62L^-^CD8^+^ T	Tfc cells express ICOS and CD40L, which interact with their corresponding ligands on B cells, and secrete IL-21, which could help B cells in the GC for Ig production. Most of Tfc cells in tonsils are effector or central memory cells. Tfc cells expressed higher level of granzyme B than CXCR5^−^ CD8^+^ T cells in tonsils and lymph nodes but not in PBMCs.	–	colorectal cancer, HIV	Human tonsils and lymph nodes tumor infiltrating Tfc cells		cytotoxic, memory
(6)	CXCR5^+^ SIV-specific CD8^+^ T	Tfc cells contribute to control of chronic SIV replication; Rapid expansion of CXCR5^+^ SIV-specific CD8 T cells is associated with enhanced control of chronic SIV infection.	-	chronic SIV infection	Tfc cells from LNs and blood of DNA/MVA vaccinated SIV-infected rhesus monkeys	-	cytotoxic, memory
(7)	CXCR5^+^PD-1^+^Tcf1^+^CD8^+^ T	Tfc cells are memory-like T cells with low expression of effector molecules, that adequately produce effector cytokines and differentiate into effector cells upon stimulation.	–	MM, CLL, DLBCL, FL	Human Tfc cells from PB and LN		cytotoxic, memory
(5)	CXCR5^+^Tim-3^−^PD-1^+^ICOS^+^CD28^+^OX40^+^CD8^+^T	Tfc cells resemble stem cells during chronic LCMV infection, undergoing self-renewal and also differentiating into the terminally exhausted CD8^+^ T cells. Tfc cells selectively proliferate after PD-1 blockade.	-	LCMV	Tfc cells from spleens of LCMV infected C57BL/6 mice	Tfc cells from CD45.2^+^ LCMV chronically infected mice are adoptively transferred into naive CD45.1^+^ recipient mice	memory
(27)	TCF1^high^Tim3^low^Blimp1^high^CD8^+^ T	Tfc cell is a less exhausted T cell population in chronic viral infection and cancer. Like stem cells, they can either maintain their phenotype or differentiate into terminally differentiated Tim3^high^ TCF1^low^ cells to maintain persistence of T cell responses.	Tcf7^loxP/loxP^; CD4-Cre (cKO), Blimp1- YFP, P14 and Ifnar1 KO P14			TCR transgenic mice recognizing LCMV	memory
(1)	CXCR5^+^CD27^+^CD28^+^CD45RO^+^CD69^+^CD7^low^CD8^+^ T	Tfc cells express CD27, CD28, CD45RO, CD69, and are CD7^low^, and produce IFN-γ and granzyme A but lack perforin, suggesting that these cells are early effector memory T cells. CD70, OX40 and ICOS are induced upon activation, and Tfc cells could secrete IFN-γ, TNF-α and IL-2. Tfc cells support survival and IgG production in tonsil B cells.	-	-	Peripheral blood and tonsil Tfc cells from healthy human		memory, assistance
(21)	CXCR5^+^IFNγ^+^PD-1^-^CXCR3^−^CD8^+^T_ab-supp_ cells	Tfc cells play an antibody-suppressor role. Tfc cells inhibit IL-4 expression by allo-specific CD4^+^ T cells, and kill allo-primed IgG^+^ B cells directly. As a result, this process improves survival of transplanted hepatocytes after transplantation.	–			C57BL/6 mice transplanted with FVB/N hepatocytes	regulation
(2)	CXCR5^+^ PD-1^+^ CD40L^+^ CD8^+^ T	Tfc cell require CD40L/CD40 and TCR/MHCI interactions to deliver help to B cells. Tfc cells contribute to the breakdown of B-cell tolerance. Tfc cells regulate the GC-B cell response and control autoantibody production.				Stat5^fl/−^ CD8^Cre/YFP^ mice	regulation
(63)	CD44^+^ICOSL^+^CXCR5^+^GITR^+^Foxp3^-^CD8^+^ Treg cells	Tfc cells reduce the numbers of Qa-1 WT donor BTLA^+^ OT-II cells to control the adoptive response. Interaction between Tfc cells and Qa-1^+^ Tfh cells inhibits production of both high affinity antibody and autoantibody.	B6. Qa-1(WT) or B6.Qa-1(D227K) mice infected with LCMV. naïve WT B6 mice, Rag2^−/−^ mice	SLE-like autoimmune diseases	Tfc cells collected from WT OT-II or B6. Qa-1 OT II mice	B6.Qa-1(WT) and B6.Qa-1(D227K) mice; naïve WT B6 mice, Rag2^−/−^ mice;	regulation
(15)	CD3^+^CXCR5^+^CD45RA^-^CD8^+^T	Tfc cells exhibit high cytotoxic activity, increased expression of IFN-γ, TNF-α, and granzymes A/B/K, and displayed antitumor efficacy *in vitro* against human follicular lymphoma cells. Tfc cells inhibit Tfh-dependent plasma blast cell differentiation.	-	follicular lymphoma	Human Tfc cells	EG7-OVA lymphoma mouse model	regulation, cytotoxic
(42)	CXCR5^+^ PD-1^high^ CD40L^+^ CD8^+^ T	Tfc cells promote B cell antibody class-switch in autoimmune disease. Allo-primed Tfc cells kill self IgG1^+^ B cells. Tfc cells express B cell costimulatory proteins, and promote B cell differentiation and Ab isotype class switching. CD8 T cells facilitate enhanced B cell expansion and Ab production in IL-2^-^ KO mice.	–	autoimmune disease		BALB/c IL-2–KO mice, scurfy and MRL/MpJ-FASlpr mice	regulation, cytotoxic
(64)	IFN-γ^+^CD40L^+^perforin^-^CD8^+^ T	Tfc cells regulate the structural integrity and functional activity of GCs in ectopic lymphoid follicles. In the absence of CD8 T cells, follicular dendritic cells disappear, production of lymphotoxin-α1β2 markedly decrease, and immunoglobulin secretion cease.	NOD.CB17-Prkdc scid/J mice (NOD-SCID) treated with anti-CD8 mAb	rheumatoid arthritis	Human Tfc cells from synovial tissue samples of rheumatoid arthritis	human synovium-SCID mouse chimeras	assistance
(65)	CXCR5^+^CD8^+^ T	CXCR5^+^CD8^+^ T cells arise in response to protein immunization and peripheral viral infection, displaying a follicular-homing phenotype, expression of cell surface molecules associated with Tfh cells and limited cytotoxic potential. CXCR5^+^ CD8^+^ T cells shape the antibody response to protein immunization and peripheral viral infection, promoting class switching to IgG2c in responding B cells.		Influenza A			assistance
(44)	CXCR5^+^ICOS^+^ CD8^+^ T	Tfc cells have deficient cytotoxicity, low IFN-γ secretion, and produce IL-4, IL-21, CXCL13. Tfc cells are capable of supporting B cell responses *in vitro*. Coculture of B cells with Tfc cells induced a twofold increase in IgG production when compared with CXCR5^-^ICOS^-^CD8^+^ T cells. ICOS is a surface marker for Tfc cell interaction with B cell. Tfc cells may be related to an unusual, CD8-mediated, antitumor reaction, mainly acting in particular cHL.		classic HL, CLL, DLBCL, FL, marginal zone lymphoma, mantle cell lymphoma,	Tfc cells from human classic HL, CLL, DLBCL, FL, marginal zone lymphoma, mantle cell lymphoma samples		assistance

Tfc: follicular cytotoxic T; SIV, simian immunodeficiency virus; HIV, human immunodeficiency virus; LCMV, lymphocytic choriomeningitis virus; MuHV, B cell-tropic herpesvirus; SLE, systemic lupus erythematosus; MM, multiple myeloma; HL, hodgkin lymphomas; CLL, chronic lymphocytic leukemia; DLBCL, diffuse large B-cell lymphoma; FL, follicular lymphoma; Gzmb, granzyme B, GC, germinal center.

Tfc cells are early memory-like cells. Genes related to Tfc cell differentiation are enriched in mitochondrial fatty acid β-oxidation, mTOR signaling and Wnt signaling, which are all related to cell maintenance and self-renewing. Moreover, it is also observed that Tfc cells are similar to CD8^+^ memory precursor cells in gene set enrichment analysis (5). Expression of Tcf1 is essential for Tfc cells maintenance and longevity. Meanwhile, over-expressions of CD62L, CD127, KLRG1 and low expressions of molecules associated to effector T cells make Tfc cells less differentiated and gain memory-like phenotype (7, 10). In addition, Tfc cells in both PB and LN express high levels of co-stimulatory molecules such as CD27 and CD28, which is compatible with their early effector memory phenotype (7).

Under specific pathogenic conditions, Tfc cells support B cell functions or assist antibody class switch through direct or indirect patterns. Tfc cells play a B cell helper function to promote virus-specific IgG production during influenza infection (43). In autoimmune diseases, increased frequency of Tfc cells leads to the breakdown of B cells tolerance and antibody over-production (2). *In vitro* experiments have shown that Tfc cells promote naïve B cells to transform into mature plasma cells secreting IgG1 antibodies. Interestingly, Tfc cells and Tfh cells are equally potent during this process, which is twice as efficient as Tc cells (44, 62). Concerning mechanism, Tfc cells facilitate antibody production from B cells *via* IL-21 and CD40L secretions (62). Moreover, *in vivo* experiment showed that after using anti-CD8 antibodies in IL-2 deficient mice, Tfc cells were eliminated, leading to a dramatic reduction of IgG1 production from GC B cells. Interestingly, Tfc cells have the potential to provide a helper-like function alone, and they also act synergistically with Tfh cells to enhance B cells differentiation, immunoglobulins production and specific class switching (42, 62). Compared to the Tfh and B cell co-culture alone, IgG1 and IgG2b secretions from B cells increase significantly when Tfc cells are added (42). Moreover, in humoral immunity, IFN-γ secreted by Tfc cells is delivered locally to B cells that localize in the T-B border and promote B cell antibody class-switching to IgG2c, and finally participate in antiviral responses (65). TCR, CD40L and ICOS are expressed on Tfc cells, and MHCI, CD40 and ICOSL are present on B cells (62, 66). It is therefore hypothesized that Tfc cells interact with B cells through the CD40L-CD40 and ICOSL-ICOS crosstalk (42). Additionally, it has been observed that in autoimmune diseases, Tfc cells assist B cells to produce IgG and IgE *via* TCR-MHCI and CD40L-CD40 interactions (2, 67).

Tfc cells perform their regulate function use either cytokines or cell-cell contact manner. Tfc cells suppress Tfh cell helper function and antibody response through ICOSL/ICOS, CD122/TIGHT and TCR/Qa-1, and inhibit antibody responses to sustain self-maintenance and suppress immunity (63). Moreover, Tfc cells selectively inhibit Tfh cells in a perforin-dependent and IL-10-independent manner, and IL-21 secreted by Tfc cells can also promote the perforin-dependent Tfh cell killing (63). In addition, Tfc cells also secrete IFN-γ, TNF-α, and granzymes A/B/K to inhibit Tfh-dependent plasma blast cell differentiation (15).

## 7 Clinical relevance of Tfc cells

### 7.1 Secondary immunodeficiency diseases

Simian immunodeficiency virus (SIV) and human acquired immunodeficiency virus (HIV) are pathogens for secondary immunodeficiency diseases in primates. Tfc cells are only observed in monkeys infected with acquired SIV, but not in the individuals that infected with SIV naturally (61). During pathogenic SIV infection, inflammatory cells infiltration and immune activation in and outside follicles are the main reasons for Tfc cell mobilization and accumulation (61). Compared to the rhesus monkeys which are under progressive stage of SIV infection, the SIV-specific Tfc cells accumulate in “SIV elite controller” monkeys, and they eliminate the infected Tfh cells in GCs efficiently. Meanwhile, in “elite controllers” the frequency of Tfc cells is negatively correlated with the peripheral viremia titer (6). The cytotoxicity of Tfc cells is mild, and their granzymes A, B, and K secretion levels are lower than those of Tc cells. In addition, Tfc cells up-regulate the expression of anti-apoptotic gene Bcl-2, which helps them to survive for a long term during SIV infection (6).

Increasing number of Tfc cells are also observed in the LNs from untreated HIV-infected patients (4, 59), and the frequency of HIV-specific Tfc cells is negatively correlated with peripheral viremia load (68). Sustained immune activation mediated by local inflammation is the main reason for HIV-specific Tfc cells expansion, while the number of Tfc cells is not related to local viral replication directly (59, 61). Cytokine productions of Tfc cells are severe impaired in HIV-infected patients, even if they highly express Gzmb and perforin, and have good cytolytic potential (3, 4, 15, 59, 61). In addition, Reuter et al. observed that in HIV patients, CD8^+^ Tc cells lose their cytotoxic capacity, while non-cytolytic Tfc cells may be responsible for the control of HIV replication. Therefore, the failure of HIV clearance may be related to the weak non-cytolytic response of Tfc cells (68).

### 7.2 B cell infection and malignant B lymphoproliferative diseases

EBV activation often occurs in immunodeficiency patients, and may leads to a variety of diseases ranging from non-malignant diseases such as infectious mononucleosis to malignancies such as lymphomas. EBV-specific Tfc cells can be detected in tonsils from patients who are currently or previously infected with EBV to control the EBV-infected B cells (7). Similar to the response in humans, on the 15^th^ day after B-cell herpesvirus MuHV-4 (murine herpesvirus 4) infection, a significant accumulation of Tfc cells was observed in the mediastinal LNs. Comparatively, in infected mice infused with Tc cells, the frequency of MuHV-4 infected B cells was 4.5 times higher than that in mice infused with Tfc cells (4).

Interactions between immune cells potentially regulate the occurrence and development of B cell malignancies. Hofland et al. (7) observed that Tfc cells are enriched in B cell follicles of HL, non-Hodgkin’s lymphoma (NHL), follicular lymphoma (FL), multiple myeloma and chronic lymphocytic leukemia patients. They may exert antitumor activity through Tfh cell suppression in a cell-cell contact manner and dose-dependent inhibition of plasma blast cell differentiation. In addition, in Tfc cell-FL cell line co-culture, CD107a expression was found to be associated with cell degranulation and tumor killing ability of Tfc cells (15).

Interestingly, Tfc cells might assist tumor proliferation in certain conditions. The ICOS-ICOSL and IL-21-IL-21R crosstalk between Tfc and R-S cells are suspected to be promotive for malignancy development. In addition, Tfc cells can be observed in tissue samples with more activation-induced cytidine deaminase(AID)^+^ B cells, which are prone to transform into spontaneous B-cell lymphoma cells (44, 69).

### 7.3 Other tumors

Tfc cells have been studied in various solid tumors such as melanoma, non-small cell lung cancer (NSCLC), pancreatic cancer, colorectal cancer and hepatocellular carcinoma (7, 58, 70, 71). Tfc cells are enriched in the TLS of tumors, but it is unknown whether they are involved in the TLS formation (59, 72, 73) In NSCLC patients Tfc cells are found both in the tumor and in the peripheral blood. When Tfc cells sorted from NSCLC patients’ tumor and blood were activated with anti-CD3/CD28, their CD107a expressions were higher than those expressed by their CXCR5^-^ counterparts (58). It is therefore suspected that Tfc cells may play a tumor-suppressive role. In addition, Tfc cells have been observed to infiltrate the tumor microenvironment of pancreatic cancer, and the frequencies of tumor-infiltrated and peripheral blood Tfc cells are positively correlated to the disease-free survival of pancreatic cancer patients. Similarly in colorectal cancer, higher frequency of Tfc cells in tumor-draining lymph nodes is related to a better prognosis of patients (74). Meanwhile, these Tfc cells express higher levels of effector genes and lower levels of genes related to cell exhaustion. Besides, the CD40L expressions on Tfc cells are positively related to the disease stage, and the levels of gzmb and perforin productions by Tfc cells decrease with disease progression (71). The frequency of Tfc cells in the peripheral blood of HBV-related hepatocellular carcinoma patients are obviously higher than that in healthy controls. Meanwhile, the Tfc cell frequency is negatively related to the HBV load and alanine aminotransferase level in these patients (75). From studies mentioned above, Tfc cells have potential anti-tumor capacity, while this effect might be affected largely by the tumor microenvironment and attenuated during disease progression.

### 7.4 Autoimmune diseases

In autoimmune diseases, Tfc cells promote B cell antibody class switching and antibody production directly, or through Tfh cells enhancement *via* cytokines indirectly (42, 62). Tfc cells assist GC-B cell tolerance and autoantibody production through CD40L/CD40 and TCR/MHCI interactions (2). Deficiency of Stat5 leads to an increase of Tfc cells, resulting in the breakdown of B cell tolerance and concomitant autoantibody production (2). In IL-2 knockout mouse model which manifests as autoimmune hemolytic anemia, Tfc cells were found to cooperate with Tfh cells to promote B cell proliferation and antibody production. When Tfc cells were depleted, the autoimmune feature of these mice mitigated and the survival prolonged due to B cell frequency reduction and decreased anti-RBC antibody production (42). Notably, affected by a variety of cytokines released during autoimmune responses including IL-21, IL-4 and IFN-γ, Tfc cells produce IL-21, which furtherly induces plasma cell differentiation and antibody class switching, constructing a positive feedback loop in autoimmune diseases (42).

### 7.5 Rejection after transplantation

The donor-specific antibodies (DSA) generation and DSA-mediated organ rejection are big challenges for organ transplantation (76, 77). Donor MHC molecules present on extracellular vesicles are recognized by alloreactive B cells, which differentiate under the help of Tfh cells to generate DSA (78). In the past decades, rejection after transplantation was regulated by T cell “depletion” or Tfh cells inhibition, however the side effects of these treatments could be severe (57, 79, 80). Zimmerer et al. (21) found that Tfc cells in hepatocyte transplant mice were located in GCs, which down-regulated the frequency of B and Tfh cells, and inhibited Tfh cell auxiliary function, reduced the generation of DSA, and finally improved the long-term survival of the graft. These Ag-specific, IFN-γ-dependent and self-MHC class I-restricted Tfc cells in the post-rejection condition up-regulated CXCR5 and down-regulated Foxp3. In addition, they may eliminate the B cells through perforin- and FasL-dependent manner.

## 8 Treatment prospects

### 8.1 Anti PD-1 therapy

Immune checkpoint blockade (ICB) is an effective treating method to maintain effective anti-tumor response by blocking inhibitory receptors on effector T cells. Thus, the emerging therapy targeting on PD-1 may restore the Tfc cell function (81). After PD-1 inhibitory pathway blockade in chronic LCMV infection mice, Tfc cells significantly proliferate and differentiate towards CXCR5^-^ CD8^+^ T cells (5). Interestingly, during chronic HIV infection, the PD-1 expression level may represent the anti-viral capacity of Tfc cells, and PD-1 inhibition decreases IFN-γ and TNF-α productions from HIV-specific Tfc cells (18). Finally, PD-1 ICB might exert anti-tumor efficacy through Tfc-modulation since PD-1 is related to Tfc cell function and localization, while the exact effects and mechanism remain to be elucidated.

### 8.2 Genetic engineering and adoptive therapy

To control viral infection effectively, researchers have tried to use Tfc cell adoptive therapy and T cell genetic engineering to increase their abundance in follicles. In Leong et al. study, Tc cells and Tfc cells were selected from chronic LCMV-infected mice, and infused into chronic LCMV-infected mice respectively. Tfc cells were found to amplify in B cell follicles efficiently, and exert stronger cytotoxic effect than Tc cells (4, 44). Ayala et al. (82) expressed CCR7 and CD62L on the surface of SIV-specific T cells through genetic engineering and infused then into rhesus monkeys. These modified T cells preferentially located in the LNs. In addition, they amplified CD8^+^ T cells with CXCR5 expression *in vitro* and infused them into rhesus monkey, and these cells were observed to enter B cell follicles effectively. They also used human CXCR5 murine leukemia virus (MuLV)-based retroviral expression vector to insert *CXCR5* gene to CD8^+^ T cells, and the expression of PD-1 was downregulated, that could be associated with the homing of remodeled-Tfc cells into B cell follicles (22). As for the potential therapeutic modalities in antibody mediated disease, Zimmerer et al. (83) found that after receiving adoptive therapy with alloprimed CXCR5^+^ CD8^+^ T cells, the alloantibody titer in kidney transplant mice was reduced, which ameliorated antibody-mediated rejection and prolonged allograft survival.

### 8.3 The application of IL-15 super agonist

IL-15 is a regulator of T cell homeostasis. Topical IL-15 super agonist ALT-803 increase the Tfc cells abundance in B cell follicles to eliminate chronic viral pathogens. ALT-803 up-regulates CXCR5 expression on Tfc cells precisely, and finally help them to localized in secondary lymphoid tissues (84). Apart from IL-15 super agonist, more stimulators are expected to enhance the function of Tfc cells.

## 9 Conclusion and perspectives

Tfc cells are an important immune cell subset with multifaceted functions in humoral immunity. There are similarities among Tfc cells and other cell types such as Tfh, Tfr or the newly identified ‘stem-like’ CD8^+^ T cells, bringing inconsistencies in identification of these cells among literatures. Deeper understanding on disparities of phenotype, transcription factors, secreted cytokines and functions between Tfc cells and other relevant T cells is needed to alleviate confusion in the field. Tfc cells eliminate infected Tfh/B cells, promote Ig secretion and regulate the B cell antibody class switch. In addition, Tfc cells have self-renewal ability and can convert to CXCR5^-^ CD8^+^ T cells in specific conditions such as chronic infection. Moreover, Tfc cells’ interactions with Tfh and B cells are complex and context-dependent. Further in-depth studies on Tfc cell transcriptome and metabolome, as well as spatial transcriptome studies on GCs/TLSs may help us further understand the whole picture of Tfc cell intrinsic signaling and their interconnections with other cells. With our deeper understanding of Tfc cell biology and more pre-clinical studies conducted in typical disease mouse models, innovative targeted therapies using or killing Tfc cells may further enlarge our arsenal towards cancer or immune-mediated diseases.

## Author contributions

YY and HH contributed to the conception of this review. YL, LR and YY prepared the manuscript. HH and BG provided expert comments. All authors contributed to the article and approved the submitted version.
